# Exosomes: Emerging Diagnostic and Therapeutic Targets in Cutaneous Diseases

**DOI:** 10.3390/ijms21239264

**Published:** 2020-12-04

**Authors:** Abdul Q. Khan, Sabah Akhtar, Kirti S. Prabhu, Lubna Zarif, Rehan Khan, Majid Alam, Joerg Buddenkotte, Aamir Ahmad, Martin Steinhoff, Shahab Uddin

**Affiliations:** 1 Translational Research Institute, Academic Health System, Hamad Medical Corporation, Doha 3050, Qatar; AKhan42@hamad.qa (A.Q.K.); KPrabhu@hamad.qa (K.S.P.); MAlam22@hamad.qa (M.A.); JBuddenkotte@hamad.qa (J.B.); 2 Department of Biological and Environmental Sciences, Qatar University, Doha 2713, Qatar; sabahaktr@gmail.com (S.A.); lz1305722@student.qu.edu.qa (L.Z.); 3 Department of Nano-Therapeutics, Institute of Nano Science and Technology, Habitat Centre, Phase 10, Sector 64, Mohali, Punjab 160062, India; rehankhan.toxic@gmail.com; 4 Dermatology Institute, Academic Health System, Hamad Medical Corporation, Doha 3050, Qatar; 5 Department of Dermatology and Venereology, Rumailah Hospital, Hamad Medical Corporation, Doha 3050, Qatar; 6 Department of Anesthesiology and Perioperative Medicine, University of Alabama at Birmingham, Birmingham, AL 35233, USA; 7 Department of Medicine, Weill Cornell Medicine Qatar, Qatar Foundation-Education City, Doha 24144, Qatar; 8 Department of Medicine, Weill Cornell Medicine, 1300 York Avenue, New York, NY 10065, USA; 9 College of Medicine, Qatar University, Doha 2713, Qatar

**Keywords:** skin, cancer, inflammation, exosomes, extracellular vesicles

## Abstract

Skin is the largest human organ and is continuously exposed to various exogenous and endogenous trigger factors affecting body homeostasis. A number of mechanisms, including genetic, inflammatory and autoimmune ones, have been implicated in the pathogenesis of cutaneous diseases. Recently, there has been considerable interest in the role that extracellular vesicles, particularly exosomes, play in human diseases, through their modulation of multiple signaling pathways. Exosomes are nano-sized vesicles secreted by all cell types. They function as cargo carriers shuttling proteins, nucleic acids, lipids etc., thus impacting the cell-cell communications and transfer of vital information/moieties critical for skin homeostasis and disease pathogenesis. This review summarizes the available knowledge on how exosomes affect pathogenesis of cutaneous diseases, and highlights their potential as future targets for the therapy of various skin diseases.

## 1. Introduction

Skin, the human body’s largest organ, provides the first line of defense against a range of external harmful biological and physical entities such as microbes, radiation, irritants or allergens. Such exposure results in a constant risk of developing cutaneous pathological conditions. According to an estimate, cutaneous diseases were the 18th leading cause of global disability-adjusted life years (DALYs) and the fourth leading cause of disability worldwide [1]. Further, because of their prevalence and severity, inflammatory, autoimmune and cancerous skin diseases pose a significant economic burden on the society. Despite major advancements in our understanding on a molecular level, skin diseases continue to impact the quality of life of millions of people worldwide. Recently, the importance of extracellular vehicles (EVs), such as exosomes, has been recognized with regards to their role in the initiation and perpetuation of various acute and chronic diseases [2,3,4,5]. EVs, including exosomes, apoptotic bodies, and microvesicles, are now considered central players in the pathogenesis and progression of cutaneous melanoma and inflammatory diseases. For instance, a number of studies have suggested EVs-mediated melanoma pathogenesis. These studies have proposed modulation of molecular mechanisms associated with cancer development in addition to angiogenesis, dysregulation of immune system and the reprograming of different genes and signaling pathways [6,7,8]. Furthermore, it has also been observed that EVs help cancer cells adapt to the fluctuating microenvironment, therapeutic challenges and drug resistance though modulation of various genetic and epigenetic events. Along this line, Peinado et al. explained how melanoma-derived EVs play major role in the formation of primary tumor and metastases by “educating” bone marrow-derived cells (BMDCs) towards a pro-vasculogenic and pro-metastatic phenotype via upregulation of the MET oncoproteins [9]. EVs also modulate the expression of non-coding RNAs (nc-RNAs) associated with the melanoma regulatory mechanisms which further supports their epigenetic activity. For example, Lunavat et al. reported that vemurafenib treatment in BRAF-mutant melanoma cells induces release of EVs with enhanced miR-211–5p expression through the involvement of microphthalmia-associated transcription factor (MITF) resulting in increased survival of parent melanoma cells [10].

Exosomes, the smallest type of EVs critical in cell communication, are nano-sized (30–120 nm) endosomal derivates present in most of the human cell types. They are composed of a lipid bilayer membrane and, upon release from the parent cells, serve as the cargo for numerous biomolecules such as nucleic acids, proteins, lipids, amino acids, metabolites and nc-RNAs. Thus, exosomes serve as major signal carrying moieties for cell-cell communications in health and disease implicated in maintaining skin development, homeostasis and disease [2,11]. Exosomes are now well known to contribute to immune dysregulation, autoimmune diseases and skin cancer development [12]. This makes exosomes a therapeutic target of importance. Modulating the composition of exosomes and the subsequent release of cargo makes them promising candidates to treat various diseases [2,11,13].

Recent updates have underlined the clinical importance of exosomes both at diagnostic and therapeutic levels. This is due to the presence of exosomes in biological fluids and their modulatory potential on various signaling pathways [14]. Befittingly, a number of clinical studies evaluating the strong immunosuppressive and regenerative effects of mesenchymal stem/stromal cells exosomes on gene delivery, regenerative medicine and immunomodulation are in progress [15]. Moreover, a number of ongoing studies are focused on the clinical importance of EVs, particularly exosomes, in cutaneous diseases [16]. Here, we highlight recent developments related to exosomes in the pathogenesis of cutaneous diseases with implications for the treatment of skin inflammation, autoimmunity and cancer.

## 2. Biogenesis of Extracellular Vesicles

**Figure 1:**
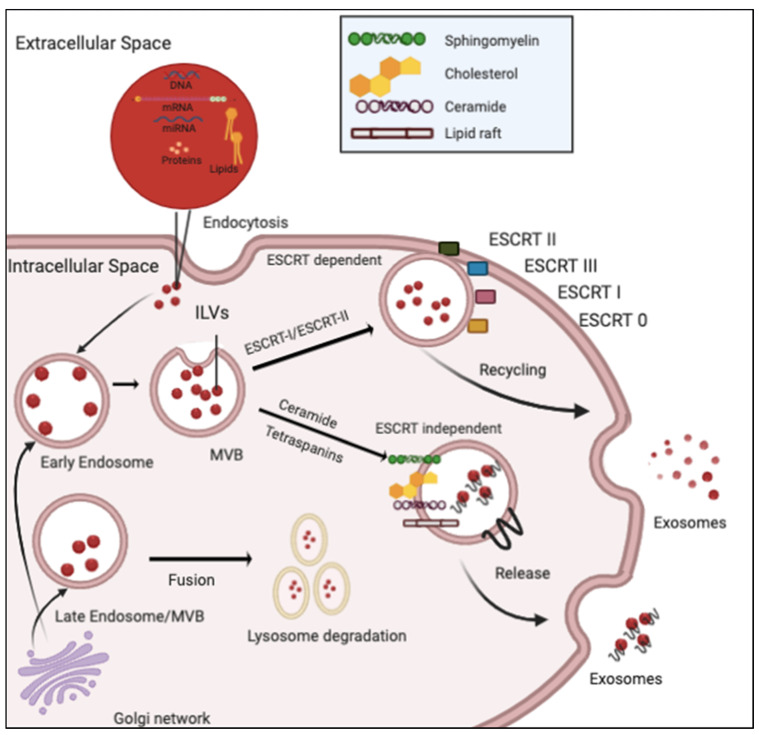
Biogenesis of exosomes: This process initiates with endocytosis. Subsequently, endocytic vesicles get formed and delivered to early endosomes which later fuse with each other forming late endosomes/multivesicular bodies (MVBs). MVBs release exosomes either by fusing with the cell membrane or subject their content to lysosomal degradation. The Trans-Golgi network (TGN) and Endosomal Sorting Complex Responsible for Transport (ESCRT) are also involved in exosome biogenesis and secretion into the extracellular space.

There are several theories regarding the formation of EVs, including exosomes. EVs are membrane-bound particles with varying features based on the size (nanovesicles, microvesicles, virus-like particles, exosome-like vesicles and microparticles), biogenesis (exosomes, membrane particles, outer membrane vesicles and shedding membrane vesicles) and specific cell origin or function (platelet-dust, oncosomes, matrix-vesicles, ectosomes, dexosomes, texosomes, epididymosomes, cardiosomes, prostasomes, rhinosomes, apoptotic bodies and tolerosomes) [17,18]. Moreover, EVs are further classified based on the type of cargo they carry and the cell type they originate from. Exosomes, with specific densities ranging from 1.13 to 1.19 g/mL, play critical roles in intracellular waste disposal and intercellular communication under physiological conditions as well as in disease pathogenesis [19]. Exosome biogenesis is a complex, multistep process involving (a) endocytic vesicle or intraluminal vesicle (ILVs) formation by invagination of the endosomal plasma membrane (during this process, proteins and cytosolic contents are engulfed); (b) generation of multivesicular bodies (MVBs) by inward budding of the endosomal membrane and (c) fusion of MVBs with the plasma membrane and release of the ILVs, called exosomes [19,20,21] (Figure 1).

Emerging evidence indicates that ILVs on maturation are sorted into endosomal-sorting complexes required for transport (ESCRT). ESCRTs are important protein machineries composed of distinct phases essential for the coordinated production such as MVB formation, vesicle budding, and protein cargo sorting [19,22]. Interestingly, an ESCRT-independent pathway critical for the sorting of exosomal cargo into MVBs has been suggested. This alternate pathway involves a ceramide-dependent process [19,21]. The complex structure of exosomes, including the presence of their biologically active contents, is often indicative of their cellular origin. It has been observed that exosomes are highly enriched for characteristic proteins like CD9, CD63, CD81, CD82 (transport, invasion and penetration of cell content); HSP70, HSP90 (antigen binding and presentation); Alix, TSG101 (exosome release) and annexins and Rab (membrane transport and fusion) [21] that are indicative of their function.

Exosomes play a vital role in a number of biological events essential for cellular homeostasis and survival. They carry biological cargo with active molecules and mediators that act in an autocrine, paracrine or juxtacrine fashion essential for skin homeostasis or, alternatively, disease pathogenesis [2]. For example, exosome contents modulate the expression or function of a number of genes in recipient cells, or regulate cell signaling pathways associated with the pathophysiology of skin diseases. Thus, exosomes serve as ideal candidates to diagnose or treat human diseases, including those that affect the skin [2].

## 3. Exosomes in Human Cancers

Exosomes are carriers of biomolecules critically involved in the pathogenesis of various human diseases including cancer, cardiovascular, neurological and autoimmune diseases [2,23,24]. Exosomes have also been shown to critically associate with the pathogenesis of various immune-mediated diseases, such as age-related macular degeneration, Sjögren’s syndrome, autoimmune uveitis and corneal allograft rejection [25]. Moreover, exosomes have been extensively investigated for their role in cancer pathogenesis and metastasis [26]. Here, we summarize the diverse role of exosomes in initiation and progression of human cancers.

### 3.1. Role of Cancer-Derived Exosomes

**Figure 2:**
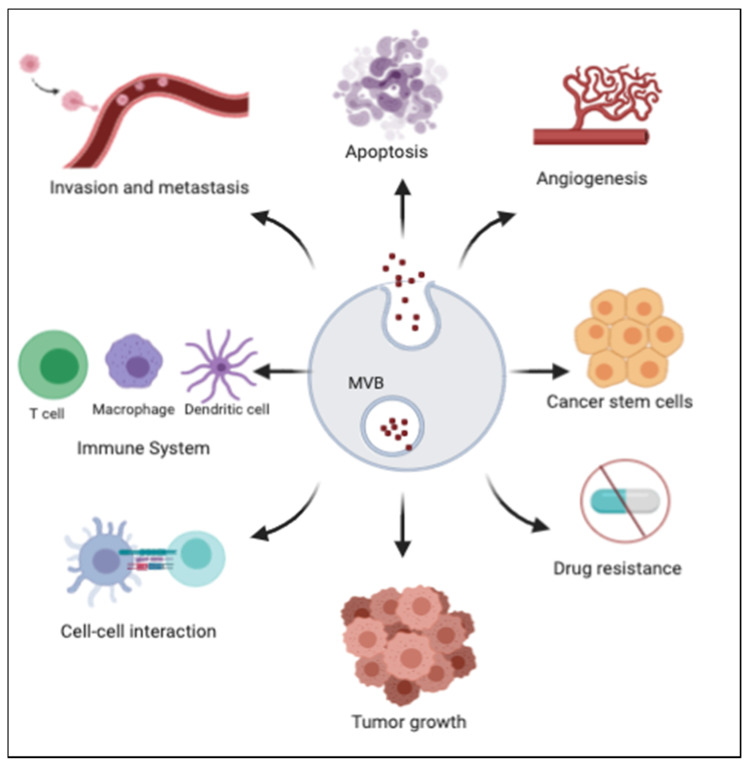
Functions of exosomes. Exosomes are involved in various functions such as tumor growth, drug resistance, angiogenesis, modulation of immune functions, cell to cell interaction and apoptosis. MVB: multivesicular bodies.

Cancer cells actively release exosomes and utilize them for tumor growth and metastasis. Exosomes carry information from one cancer cell to another, or between normal and cancer cells thereby contributing to cancer development and metastasis. As an evidence supporting their diverse functions, particularly relevant to maintaining homeostasis, exosomes can help induce immune defense mechanisms to combat tumor progression by activating immune mechanisms that induce tumor cell apoptosis or modulate autophagy [3,27,28,29,30]. Through the delivery of their cargo contents to recipient cells, exosomes alter multiple cell functions with profound effects on metastasis, angiogenesis and immunosuppression (Figure 2).

### 3.2. Role of Tumor Derived Exosomes (TDEs) in Cell-Cell Communications

Tumor derived exosomes (TDEs) play an important role in cancer metastasis by altering tumor microenvironment (TME) through modulating cell-cell communication between TDEs and distant host cells [31]. For example, exosomes derived from epithelial carcinomas contain epithelial cell adhesion protein (EpCAM) and CD24 which, upon release, are associated with increased cancer aggressiveness [32]. TDEs also cargo some pro-angiogenic factors such as vascular endothelial growth factor (VEGF), IL-8, matrix metalloproteinase 9 (MMP9) or hepatocyte growth factor, secreted by stromal cells, thereby inducing endothelial cell proliferation and facilitating tumor angiogenesis [32]. Additionally, TDEs play an important role in the modulation of extracellular matrix proteins during tumor development. Owing to being rich in proteases, TDEs help degrade extracellular matrix proteins by degrading collagen, fibronectin or laminins. The modulation of extracellular matrix has serious effects on tumor cell motility, adhesion and invasiveness by promoting migration of not only tumor cells, but also of immune cells [33].

### 3.3. Role of Tumor Derived Exosomes in Immunity

There are various complex immunological roles of exosomes in cancer which include polarization of cancer immunity or alteration of tumor antigen presentation [34,35]. Owing to their functional heterogeneity, the pro- and anti-tumorigenic roles of exosomes in humans are still very poorly understood. Although implicated in inducing anti-tumor immune responses, exosomes may cause immune evasion due to altering IL-6 expression levels in dendritic precursor cells of bone marrow (BM) leading to impaired maturation of dendritic cells [36]. Thus, exosomes are involved both in pro- as well as anti-tumor immune responses which needs to be considered when designing therapeutic strategies for cancer [34].

#### 3.3.1. Anti-Tumorigenic Immune Responses

TDEs are capable of activating natural killer (NK) cells through HSP70 protein production. They also provide tumor-derived antigens that are presented to activated T cells thereby inducing tumor defense mechanisms [37,38,39,40]. Moreover, tumor-associated immune defense responses, including induction of apoptosis, are mediated by exosomes due to release of pro-inflammatory mediators from dendritic cells, macrophages or T cells [41]. For example, immune cell-derived exosomes mediate immune responses through tumor necrosis factor alpha (TNF-α) production and release, which can subsequently permit epithelial cells to secrete more pro-inflammatory cytokines such as additional TNF-α, IL8 and RANTES [42]. Also, dendritic cells-derived exosomes induce anti-tumor responses in NK cells via exosome-mediated pathways [43].

#### 3.3.2. Pro-Tumorigenic Immune Responses

TDEs play an important role in TME as they disrupt immune surveillance and stimulate intercellular communication in various immune cells [34]. For instance, TDEs inhibit NK cell function by decreasing their cell number and activity within the TME [44,45]. NK cells are the major cytotoxic cells of the immune system that contain both inhibitory and activating receptors and functions in immune surveillance. It is well known that distorted regulation of NK cells and its receptors plays major role in disease pathogenesis [46]. Natural killer group-2 member D (NKG2D) receptor is an activating cell surface receptor usually expressed in cytotoxic immune cells and is most abundant in NK cells. It is also expressed by various T cell types including NKT cells, CD8+ T cells, and γδT cells. Interestingly, under normal conditions, NKG2D receptors are in inactive state but get activated to perform immune surveillance and cytotoxic actions due to ligand (encoded by multiple genes) binding in response to pathogen and stress etc. [46]. Distortions in gene expression of the NKG2D ligands due to various factors can lead to evasion of immune surveillance and disease pathogenesis [46,47]. In melanoma, exosomes mediate immune suppression and facilitate metastasis. Melanoma-derived exosomal PDL-1 mediates immune suppression through inhibition of CD8^+^ T cell function, thereby permitting tumor growth. Accordingly, melanoma patients show enhanced levels of exosomal PDL-1, which positively correlates with melanoma metastasis indicating a pro-tumorigenic role of exosomes in immune suppression [48,49,50].

### 3.4. Role of TDEs in Angiogenesis

TDEs play an essential role in inducing angiogenesis and thereby tumor growth. They carry mRNAs and angiogenic proteins that cause endothelial cell proliferation in the TME. For example, glioblastoma cells-derived exosomes contain angiogenic proteins, miRNA or mRNA and communicate with healthy cells in host tissue [51]. Exosome-induced endothelial cell proliferation has also been revealed in colorectal cancer cells-derived exosomes containing cell cycle-associated mRNAs that participate in proliferation of endothelial cells, thereby inducing angiogenesis and causing tumor growth and metastasis [52]. The exosomes derived from the cells of glioblastoma multiforme cause angiogenesis in vitro and ex vivo through phenotypic changes in endothelial cells, when these exosomes are grown in hypoxic conditions [53].

### 3.5. Role of TDEs in Migration, Invasion and Metastasis

A role of cancer cell derived exosomes in remodeling of extracellular matrix and conversion of benign epithelial cells into malignant cells has been suggested [54]. Thus, exosomes contribute to epithelial- mesenchymal transition within the TME, as well as invadopodia formation and reorganization of cytoskeleton which contributes to tumor development. Exosomes derived from epithelial cells differ from mesenchymal cell-derived exosomes, and this heterogeneity of exosomes affects therapy resistance and metastasis. Endothelial cells are connected through tight junctions and adherens junctions which maintain their vascular barrier functions during tissue homeostasis. However, during tumorigenesis, TDEs disrupt these junctions leading to increased vascular permeability and tumor invasion [9,55,56].

In summary, EVs, particularly exosomes, carry information from one cancer cell to another, or between normal and cancer cells. Thus, they contribute to cancer development, metastasis or induce immune defense mechanisms to combat tumor progression by activating immune mechanisms that induce tumor cell apoptosis or modulate autophagy.

## 4. Extracellular Vesicles in Skin

### 4.1. Skin Melanocytes and Melanosomes

Melanocytes are the melanin-producing heterogenous group of cells, derived from embryonic neural crest cells, present in skin epidermis and hair follicles. These cells are also present in the middle layer of the eye, meninges, bones, inner ear, vaginal epithelium nervous system and heart [57] and execute different functions via targeting a range of signaling mechanisms though they have the common origin [57].

**Figure 3:**
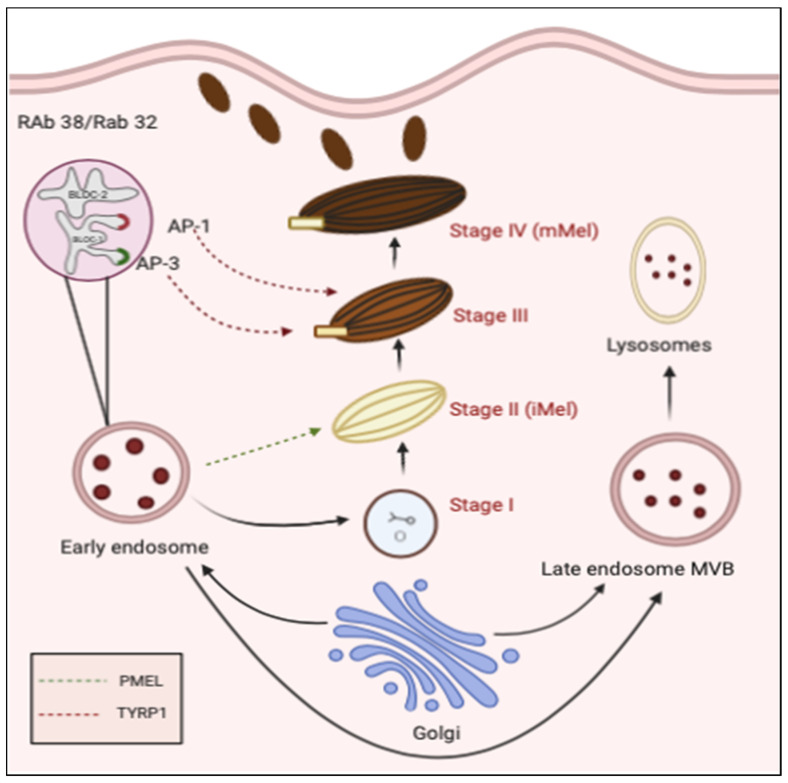
Schematic representation of melanosome biogenesis in melanocytes. Immature melanosomes stage I is derived from early endosomes. Stage II (immature melanosomes—iMel) is formation of non-pigmented melanosomes containing PMEL fibrils (indicated in green dotted lines). At stage III, melanogenesis begins with translocation of tyrosinase related proteins (TYR—indicated in red dotted lines) from tubular elements of early endosomes. Stage IV marks the maturation of melanosomes (mMel) facilitating melanin deposition. Adaptor protein-3 (AP-3) and Adaptor protein-1 (AP-1) are adaptors for sorting TYR to melanosomes. BLOC-1 and BLOC-2 are regulators of endosome-to-melanosome transport. Rab32/38 are tissue specific proteins which play essential roles in pigmentation.

Melanocytes paly critical role in skin pigmentation and protection from exogenous and endogenous substances and hence are critical in skin homeostasis. For instance, melanin produced by these cells prevents cutaneous cellular DNA damage from harmful UV radiations. Furthermore, melanocytes secrete a special type of endosomal organelles, termed as melanosomes, which are approximately 500 nm in diameter. These melanosomes are the principles sites for melanin production and hence are the major organelles that protect from harmful exposures [58]. Production of melanosomes in melanocytes is a complex phenomenon regulated at both genetic and protein level in response to various stimuli [59]. Considering the critical role of melanosomes in the pathophysiology of cutaneous pigmentation disorders including melasma and vitiligo, it is important to understand the biogenesis and transfer of melanosomes within and outside of the skin structures [58]. In this line, various studies suggest that melanosome formation and transfer is a multistep phenomenon. It includes formation of nonpigmented pre-melanosomes (stage I), acquisition of internal striations (stage II), deposition of the melanin pigment (stage III), and eventually formation of the mature melanosomes (stage IV) [59,60] (Figure 3). Moreover, transfer of melanosomes is also a multistep process that includes direct transfer of melanosomes into keratinocytes, uptake of melanosomes from melanocytes into keratinocytes and through partial cytophagocytosis of melanocytes by adjacent keratinocytes [58,59,61].

### 4.2. Skin Pathobiology

Skin, being the largest organ, is crucial for protection and survival. Hence, it is prone to various cutaneous aliments including cancer and autoimmune diseases. Structurally, skin consists of three major layers, namely, epidermis, dermis and hypodermis. In addition, it has various supporting structures like hair follicles, eccrine sweat glands, sebaceous glands and apocrine glands. Keratinocytes are the major cellular components of epidermis among the melanocytes, Langerhans cells, and Merkel cells [62]. A number of reports have supported involvement of different molecular, cellular and histological changes in cutaneous pathogenesis [63,64,65]. Development of cutaneous malignancies such as nonmelanoma (basal cell carcinoma, squamous cell carcinoma) and melanoma is a complex and heterogeneric phenomenon. This is due to accretion of genetic and epigenetic changes that ultimately result in the deregulated expression and functioning of molecular entities critical in biological homeostasis including tumor suppressor genes as well as oncogenes. A number of physical (e.g., environmental toxicants, radiation etc.) and biological (e.g., viruses such as human papilloma virus) exposures have been recognized as the major etiological factors. Furthermore, these factors cause skin cancer pathogenesis by inducing DNA damage, over-production of ROS and inflammation causing aberrant expression of genes associated with homeostasis, reprogramming of stemness and metabolic pathways [66]. Moreover, like other body parts, skin has a well-organized defense mechanism at molecular cellular and histological levels. However, these defense mechanisms get distorted due to continuous exposure to physical and biological hazards. At signaling level, several mechanisms get dysregulated, including activation of survival pathways such as JAK/STAT3, NF-κB, and downregulation of apoptotic pathways.

Inflammation is a pivotal underlying mechanism associated with defense and immunity but may also lead to disease pathogenesis if deregulated. Chronic inflammation plays a critical role in the development of most of the cutaneous diseases, including cancer, seborrheic dermatitis, acne, collagen vascular disorders, atopic dermatitis (AD) and autoimmune diseases such as psoriasis. Moreover, various studies have explored the role of deregulated expression and secretion of various inflammatory mediators including cytokines and chemokines which are critically associated with the pathogenesis of cutaneous diseases [67,68]. Skin autoimmune diseases, due to a range of genetic, epigenetic and environmental factors, represent another major skin problem with increasing therapeutic challenges. They require deep and through investigation to further understand the underlying mechanisms. Due to deregulated production of autoantibodies, cutaneous autoimmune diseases trigger a number of molecular and cellular alterations leading to distortion of skin barrier and, ultimately, wounds or blisters [69]. Chronic and deregulated functioning of immune cells, particularly T cells, has been widely reported in the pathobiology of various cutaneous aliments including the autoimmune problems. Considering the vital role of T cells, cutaneous autoimmune diseases can be categorized as T1 cell-mediated (such as, vitiligo), T2 cell-induced (such as, atopic dermatitis), T17/T22 cell associated (such as, psoriasis) and Treg cell–dominated (such as, melanoma). Hence, presence of specific T cell types induces cellular and molecular alterations associated with the development of specific cutaneous pathologies [70]. In a nutshell, it can be said that pathogenesis of cutaneous disease involves a number of genetic and epigenetic changes which result in deregulated expression and functioning of major molecular and cellular regulatory mechanisms converging towards disease pathogenesis.

## 5. Exosomes and Reprogramming in Skin Cancer Pathogenesis

Over the years, it has been realized that reprograming at genetic level is critical for the expression of various molecules to support disease pathogenesis. Cellular reprograming is the phenomenon to revert the mature cells into induced pluripotent stem cells (iPSCs) or the conversion of somatic or differentiated ells into iPSCs through altered expression of transcription factors Oct4, Sox2, Klf4 and c-Myc [71]. Exosomes play a major role in cancer development and metastasis by delivering peptides, proteins, proteases, lipids or RNA as functional mediators to allow cell-cell communication and control cell function during tumor progression and metastasis [72]. They can mediate tumor cell reprogramming by modulating expression and functioning of genes or proteins associated with various steps of cancer metastasis such as invasion, vessel formation and cell survival. Exosomes also control attachment of tumor cells to vascular endothelium via released proteases indicating that exosomes affect tumor cell adhesion to blood vessels thereby increasing tumor infiltration and metastasis [73,74]. Consequently, manipulating the release of mediators from exosomes may lead to suppression of tumor-promoting, receptor-mediated cell signaling with potential therapeutic implications [72]. In addition, exosomes derived from normal and cancer cells have been shown to affect unique cellular and molecular events in the recipient cells via targeting unique gene expression. For instance, insertion of cancer or normal cell derived exosomes in primary human normal oral keratinocytes (HNOK) altered molecular changes involved in matrix modulation, cytoskeletal remodeling, anti-inflammatory action, deubiquitination, lipid metabolism and membrane trafficking. Only cancer cells-derived exosomes were shown to induce transcriptional reprogramming in recipient cells to mediate angiogenesis, immune evasion/modulation, cell fate alteration and metastasis [75].

Melanoma cells produce a number of EV subtypes, including shedding vesicles and exosomes which affect progression of melanoma via triggering various signaling mechanisms associated with melanoma proliferation and metastasis [8]. Accordingly, multiple reports confirm that exosomal content such as nc-RNAs, lipids, deregulated signaling proteins critically affect melanoma progression via regulating cell reprogramming, immune function and maintenance of TME. For example, melanoma-derived exosomes play a central role in reprograming of normal fibroblasts into cancer-associated fibroblasts (CAFs) thereby promoting tumor progression [76]. In particular, transition of normal fibroblasts into CAFs involves reprogramming by exosomal delivery of lncRNA Gm26809 into normal fibroblasts [76]. This leads to increased expression of CAFs markers (α-SMA and FAP) and Gm26809 knockdown inhibits tumor metastasis features. Therefore, targeting exosomes with Gm26809 may provide a novel therapeutic tool for melanoma therapy.

Cancer stem cell (CSCs)-derived exosomes regulate critical signaling mechanisms related to tumor growth, TME and extracellular matrix regulation, as well as tumor metastasis. Furthermore, exosomes secreted by CSCs and/or mesenchymal stem cells activate signaling pathways associated with drug-resistance, tumor progression and metastasis [77]. Interestingly, exosomes, through epithelial-mesenchymal transition (EMT) modulation or by regulating cell stemness, modulate cell signaling pathways, including Wnt, Notch, and Hedgehog pathways, which maintains the balance between non-CSCs and CSCs in tumors [78]. A recent study revealed that mesenchymal stem cells associated exosomes trigger differentiation of myeloid cells into immunosuppressive M2-polarized macrophages in order to mediate metastasis via increased PD-L1 overexpression [79]. Further, another investigation demonstrated that CSCs-derived exosomes potentiate EMT of normal epithelial cells and coordinate with distant cells to trigger cancer pathogenesis via modulating the expression of nc-RNAs [80]. Moreover, it has been also observed that exosomes from bone marrow-derived mesenchymal stem cells trigger cancer metastasis via nc-RNA-induced activation of STAT3 signaling related with EMT [81]. The role of EMT and STAT3 in tumorigenesis is well established [82,83] and therefore their modulation by exosomes needs to be investigated in detail.

In squamous cell cancer and keratinocyte-derived skin tumors, stem cell-derived exosomes modulate tumor growth and cell proliferation. Kim et al. demonstrated that exosomes isolated from iPSC-derived mesenchymal stem cells (iMSCs) enhance tumor growth and cell proliferation in a human keratinocyte tumor cell line (HaCaT) via phosphorylation of extracellular signal-regulated kinase (ERK)-1/2, indicating potential clinical importance of iMSC-derived exosomes [84]. In squamous cell carcinoma, exosomes generate and release mediators that promote tumor metastasis through reprogramming of metastatic signaling molecules in various cells such as fibroblasts, endothelial cells and BM progenitor cells [9]. Bone marrow-derived cells have been shown to play critical role in cancer pathogenesis via affecting metastasis, migration and malignant conversion [85]. Various reports suggest that cancer cells initiate pre- metastasis or niche establishment via secreting various factors and educating or reprogramming bone marrow derived hematopoietic progenitor cells in order to establish their colonization at distant sites [86,87]. For instance, bone marrow derived hematopoietic progenitor cells with VEGFR-1 expression play central role in the initiation of the pre-metastatic niche [85]. Met expression in exosomes attenuates metastatic behavior of bone marrow cells indicating exosome generation triggers metastatic features of bone marrow cells [9,88].

In summary, the available data suggests that EVs, including exosomes, modulate epigenetic and genetic events in cutaneous diseases (e.g., melanoma) to reprogram cell functioning in order to adapt to the fluctuating microenvironment and therapeutic challenges. Hence targeting these events may provide therapeutic benefits in skin diseases. However, the information regarding mechanisms by which exosomes promote skin pathogenesis is still sparse and more studies are needed.

## 6. Exosomes in Melanoma Pathogenesis

For various cancers, including melanoma, it has been demonstrated that exosomes release important mediators into the extracellular space in order to promote tumor growth and metastasis [6,9,89,90,91]. Further, exosomes have been shown to fuse with target cells thereby contributing to cancer pathogenesis [21,92]. Notably, keratinocytes are capable of releasing specific miRNAs (e.g., miR-203, hsa-miR-3196) targeting melanocytes, which subsequently alter melanogenesis by upregulating genes involved in melanogenesis such as GADD45B [93,94]. Along that line, Chen et al. demonstrated that these mediators, particularly microRNA (e.g., miR-300) released by exosomes, may cause melanoma initiation via targeting growth arrest and DNA-damage-inducible beta (Gadd45b) expression, an important gene associated with cell protection, both in vitro and in vivo [95]. Melanocytes contain melanosomes which produce melanin pigment. Released melanin products have been shown to modify communication between melanoma cells and its microenvironment by controlling genes involved in cell proliferation, inflammation. For example, abnormal production of various miRNAs like −149, −211, −23, −let7a and −let7b activate exosomes, thereby stimulating the WNT pathway which ultimately leads to development of metastatic phenotype of invasive melanoma cells [96]. In addition, certain oncoproteins such as MET oncoprotein get accumulated inside melanoma-derived exosomes, and fusion of these exosomes with fibroblasts stimulates chemotaxis and inflammation by upregulating Src and S100 [56]. Exosomes also contribute to melanoma-immune cell communication. They are key mediators of inflammation and immune response. Additionally, they alter the activities of immune cells thereby regulating immune responses and modulation of anti-tumor activity of body’s immune cells in melanoma and other cancer types [97,98].

If exosomes-induced inflammation is uncontrolled and inadequately settled, it gets converted into chronic inflammation which associates with an enhanced risk of malignant cell transformation, including melanomas [99,100]. For example, Gerloff et al. demonstrated that exosomes derived from melanoma cells and taken up by THP-1-derived macrophages induce tumor-promoting tumor-associated macrophages phenotype with increased expression of genes associated with inflammation, chemotaxis and angiogenesis [101]. Another mechanism of exosome-induced inflammation in the pathogenesis of melanoma involves the ability of exosomes to stimulate cell proliferation, cell survival, chemotaxis or activate monocytes to release proinflammatory cytokines such as C-C motif chemokine ligands (CCL2, CCL4), IL-6 and IL-10 to induce activation of B cells [102,103] which subsequently promotes deregulated immune functions.

Notably, with respect to future planning of therapeutics, exosomes may also release mediators that facilitate anti-tumoral capacities. For example, melanoma derived exosomes exert anti-tumor effects by transferring tumor antigens from cancer cells to dendritic cells, thereby inducing CD8+ T-cell dependent anti-tumor effects in human melanoma cells ex vivo and in mouse as well as when exosomes are fused with bone marrow-derived myeloid precursor cells (CD11b^+^Gr-1^+^) and injected into mice with established melanoma [38,104]. Another anti-tumor effect of exosomes was noted when monocyte-derived dendritic cells were pulsed with exosomes for the stimulation of T lymphocytes. These cross-present mart-1 antigen to cytotoxic T-lymphocytes resulting in their antigen-specific expansion in melanoma patients [104,105,106].

Exosomes are also involved in immune-endothelial cell communication in melanoma contributing to angiogenesis and hypoxia. For example, exosomes derived from melanoma are capable of inducing expression of pro-angiogenic and inflammatory genes including hypoxia-inducible factor 1 alpha (HIF-1α) and TNF-α in lymph nodes. Enhanced expression of these genes suggests participation of M2-macrophages in the pathobiology of melanoma-associated hypoxia and inflammation, which may contribute to melanoma metastasis [107]. Recently, Bardi et al. [108] demonstrated a key role for exosomes in melanoma growth, survival and metastasis wherein tumor-associated activation of macrophages plays an essential role, as demonstrated by functional studies and qPCR in-vitro. They further explored that exosome- mediated activation of mixed M1 and M2 macrophage populations results in macrophage activation phenotypes that are pro-tumorigenic [91,109].

With respect to exosome-induced inflammation in melanoma, Peinado et al. [9] demonstrated that tumor-derived exosomes from patients with advanced and metastatic melanomas carry enhanced levels of various proteins like Rab27a, TYRP2, MET, VLA4 and HSP70, many of which are related to ECM remodelling and inflammation. These exosomes are then exuviated by tumor cells into the extracellular surroundings where they are subsequently transported to the bone marrow, lymphatic organs, lungs, or other sites of metastasis. At these destinations, exosomes get engaged in the formation of tumor ‘niches’ by inducing tissue inflammation. Moreover, in a mouse model of melanoma, it was demonstrated that intravenously-administered exosomes from a metastatic mouse melanoma cell line (B16-F10) boosted both melanoma invasion and metastasis [9]. These exosomes co-localized with melanoma cells inside the lung and lymph nodes where they promoted vascular permeability and pro-inflammatory conditions resulting in metastasis [9,110]. Before occurrence of tumor metastasis, melanoma-derived exosomes are also capable of recruiting bone marrow-derived cells at pre-metastatic sites by upregulation of pro-inflammatory factors (e.g., S100), thereby enhancing melanoma invasion and metastasis [111,112].

In another report stressing the role of exosome-regulated inflammation in melanoma, it was stated that melanoma exosomes determine endothelial tubule morphology and induce endothelial spheroid production, while simultaneously eliciting paracrine endothelial cell signaling by upregulation of certain cytokines (e.g., L-1α, FGF, GCS-F, TNF-α) involved in tumor-associated inflammation [113]. Moreover, in another study it was demonstrated that exosome-mediated conditioning of lymph nodes in metastasis of melanoma cells takes place as a result of melanoma cell recruitment, extracellular matrix deposition and vascular proliferation in lymphatic tissues through synchronized molecular signals involved in inflammatory responses [113,114]. Marton et al. [115] studied the inflammatory and immunomodulatory effect of exosomes deriving from melanoma cells, and quantified the activation of NF-κB as well as production of cytokines (IFN-γ and IL-16) and chemokines (IL-8, and MCP-1 or CCL2) involved in inflammation and maturation of macrophages. These cytokines and chemokines were previously established as regulators and mediators of anti-cancer responses [115]. Thus, activation of NF-κB in macrophages may lead to immune activation and inflammatory cytokines gene transcription and upregulation of CCL2 and MIP-2, the mediators that have been shown to be involved in tumor-associated inflammation, angiogenesis and tumorigenesis [116,117]. Bardi et al. [108] have recently studied the dependency of melanoma pathogenesis on inflammatory processes and hypothesized that exosomes may carry inflammation-related mRNA content. Using primary melanocyte exosomes as control small extracellular vesicles, the authors detected inflammation-related melanoma exosome mRNA contents by RT-qPCR array, as well as upregulation of proangiogenic mediators (e.g., CXCL1, CXCL2, and CXCL8) [108]. These results support a close crosstalk between immune cell-derived inflammatory signals (e.g., chemokines) and tumor cells. They also suggest that mRNA content in various melanoma exosomes can aid in exosome categorization in terms of their inflammation-associated pro-tumor attributes.

Another recent study indicates an implication of micro RNA-155 (miR-155) from murine and human melanoma-derived exosomes in vitro. As miR-155 was closely associated with inflammation and immune responses [118,119], it was concluded that targeting of miR-155 may be a potential novel strategy to treat melanoma. A role for miR-155 was also implicated in the CAFs-supported progression of melanoma cells, via the regulation of JAK2-STAT3 pathway through modulation of VEGF, FGF, and MMP9 [120].

In light of the above discussion, it can be concluded that EVs, such as exosomes, are one of the major carriers of biomolecules including oncoproteins, nc-RNAs, cytokines, chemokines, interleukins critically associated with melanoma pathogenesis.

## 7. Exosomes in Non-Melanoma Skin Cancer (NMSC)

EVs also play major role in nonmelanoma skin cancer via modulating various factors associated with cancer hallmarks including reprogramming. As an example, squamous cell carcinoma (SCC) derived EVs, with enhanced desmoglein 2 (Dsg2) expression, have been shown to promote cancer pathogenesis via targeting survival pathways and TME [121]. Moreover, exosomes play critical role in cancer therapy as supported by a study which reported that exosomes are critical in 5-aminolevulinic acid photodynamic therapy (ALA-PDT) of cutaneous SCC. According to this report, exosomes mediate their antitumoral action via inducing dendritic cell maturation and fibroblast secretion of TGF-β1, thus providing a novel strategy for anti-tumor immune response [122]. It is worth mentioning that an initial investigation regarding the role of exosomal content, particularly nc-RNA in the metastasis of basal cell carcinoma (BCC), has shown some promising results [123]. In recent years, nc-RNAs have emerged as potential targets for diagnosis as well as therapy [124]. Considering these indications for an important role of EVs in NMSC pathogenesis, more mechanistic studies are urgently needed.

## 8. Exosomes and Cutaneous Chronic Inflammatory Conditions

A majority of skin diseases are inflammatory in nature. Inflammation is part of the body’s immune defense mechanism. However, uncontrolled inflammation leads to chronic inflammatory skin diseases such as AD or psoriasis [125]. Here, we focus on exemplary evidence as to how exosomes contribute to the initiation and perpetuation of chronic skin inflammation. Studies show that exosomes play a critical role in the pathogenesis of inflammatory skin conditions and cutaneous autoimmune diseases. For example, psoriasis is a chronic-relapsing autoimmune inflammatory skin disease, characterized by increased infiltration of CD4+ and CD8+ T cells, neutrophils, NK cells, NKT cells, mast cells, macrophages, and type-1 and type-3 innate lymphoid cells [126]. Initially, it was speculated that TH1-derived exosomes transport cytokines such as TNF to neighboring antigen-presenting dendritic cells thereby promoting early development of psoriasis. However, new evidence indicates a more primary role of TH-17 cells and IL-17 (deriving not only from Th17 cells) in the pathophysiology of this common disease. IL-17 release is partly under the control of IL-23 which is released in exosomes by dermal dendritic cells [126,127]. Because anti-IL-17 and IL-23 targeted therapy has been demonstrated as particularly effective, one might assume that manipulating exosome function from dendritic cells in psoriasis may be a promising avenue to combat this frequent chronic disease. Along that line, Jiang et al. [128] demonstrated that exosomes, isolated from psoriasis-like keratinocytes and treated with a ‘psoriatic cytokine cocktail’ (involving IL-17A, IL-22, and TNF-a), are critical players in the induction of psoriatic inflammation, due to T-cell and neutrophil activation and infiltration. An enhanced expression of inflammatory cytokines IL-6, IL-8, and TNF-α as well as activated NF-κB or p38 MAPK cell signaling in neutrophils, after stimulation with keratinocyte-derived exosomes, was reported, suggesting that controlling keratinocyte-derived exosomes may have a therapeutic potential to treat psoriasis [128].

Interestingly, it has also been demonstrated that IFN-γ can mediate exosomal secretion of HSP90 in HaCaT cells which subsequently plays a critical role in psoriasis pathogenesis by activating innate and adoptive immune cells such as dendritic cells, lymphocytes, neutrophils, NK cells and macrophages [129,130]. As a proof-of-principle therapeutic approach, it has been shown that inhibiting IFN-γ expression and exosome secretion of HSP90 may alleviate psoriasis [129]. Further, elevated phospholipase A_2_ (PLA_2_) activity linked to lipid-specific T cell inflammatory skin responses results in elevated levels of PLA_2_ products, including prostaglandin E_2_ (PGE_2_), PGF_2α_, and 12-HETE (12-hydroxyeicosatetraenoic acid) in patients with psoriasis associated with metabolic syndrome. It has been shown that PLA_2_-responsive CD1a-reactive T cells are elevated in psoriasis patients. Furthermore, it was also noted that cytosolic phospholipase PLA2G4D was expressed in mast cells and keratinocytes transported by exosomes [126], thereby contributing to psoriasis pathophysiology

A body of evidence suggests that nc-RNAs transported by exosomes play a critical role in the regulation of inflammatory responses against endotoxin and also in various diseases including psoriatic arthritis [131]. Recently, it has been shown that plasma exosomal microRNAs are critical in pathogenesis of autoimmune diseases including psoriasis and may be used as disease or prognostic ‘biomarkers’ [132]. In particular, this study showed the role of plasma exosomal micro-RNAs, particularly hsa-miR-151a-3p, hsa-miR-199a-5p, hsa-miR-370-3p, hsa-miR-589-5p, and hsa-miR-769-5p in the development of both immune diseases and bone metabolic dysregulation in psoriatic arthritis (PsA), psoriasis vulgaris (PV), rheumatoid arthritis (RA) and gouty arthritis (GA) patients [132]. Recent evidence also indicates a critical role of nc-RNAs (both miRNAs and lncRNAs) transported via exosomes to target cells in the pathophysiology of psoriatic arthritis, and these nc-RNAs may thus be potential new ‘biomarkers’ for its management [133]. Moreover, Fatima et al. documented the potential of ncRNAs in mesenchymal stem cell-derived EVs in tissue repair related to cutaneous injuries by regulating various mechanisms including matrix remodeling, EMT, attenuation of inflammatory mediators and modulation of immune status [134].

There is some data supporting the role of EVs, particularly exosomes, in the amelioration of burn and associated complications as well. For example, Li et al. demonstrated that human umbilical cord mesenchymal stem cells (hUCMSC)-derived exosomes, that overexpress miR-181c, attenuate burns-induced inflammation in rats or in macrophages, via downregulating TLR4 associated signaling mechanisms including NF-κB/p65 activation [135]. Moreover, MSC-derived exosomes promote skin flap recovery in rats via alleviation of inflammation, growth and neovascularization [136]. Exosomes released from human dermal adipocytes displaying overexpression of circRNA, has- circ0075932, in burned skin of obese persons have been shown to promote inflammation and cell death in dermal keratinocytes via modulating NF-κB [137]. This indicates a probable underlying mechanism of slower healing in obese individuals.

## 9. Role of EVs (Exosomes) in Skin Autoimmune Diseases

There are various reports suggesting clinical importance of EVs in AD, an inflammatory skin disease with increasing prevalence due to microbial infections with enhanced production of EVs. A recent report on the subject revealed that the circulating CD3+HLA-DR+ EVs can be used as markers for T cell- associated pathological conditions including AD [138]. Moreover, it has been also observed that Lactobacillus plantarum-derived membrane vesicles (MVs) attenuate AD pathogenesis due to microbes-associated EVs, thus indicating their therapeutic importance [139]. There have also been some reports demonstrating the important role of MVs in the pathogenesis of AD due to Staphylococcus aureus. The suggested mechanisms included transfer of crucial microbial proteins and induction of inflammatory responses in the host cells, accompanied by increased pro-inflammatory cytokines/chemokines, Th1, Th2, and Th17- induced inflammatory changes, endothelial cell activation and monocyte recruitment [140,141,142]. Interestingly, disruption of microbial MVs release has been found to alleviate AD pathogenesis [141]. Therapeutic importance of EVs has been also elucidated in contact hypersensitivity via inhibition of cytotoxic T cells and T helper cells and regulatory T cells [143]. Further, human adipose tissue-derived mesenchymal stem cell-derived exosomes (ASC-exosomes) attenuate AD pathogenesis via modulating IgE, eosinophils, infiltration of CD86+, and CD206+ cells, inhibition of inflammatory cytokines such as interleukin (IL-4, IL-23, IL-31, and TNF-α) and hence suggests therapeutic importance of ASC-exosomes [144].

Role of EVs, including exosomes, in systemic lupus erythematosus (SLE), another major autoimmune disease that affects skin and other organs, has been well investigated [145]. Interestingly, it has now become evident that circulating EVs including exosomes play major role in SLE and can potentially be exploited as clinical biomarkers for SLE [146,147]. Furthermore, nc-RNAs contents of circulating exosomes has also been found to play a major role in SLE pathogenesis and hence could be of therapeutic importance as well [148,149]. Finally, EVs, including exosomes, has been proposed as attractive therapeutic targets for managing hair follicles-related cutaneous ailments as they regulate and carry a number of components vital for stem cell functioning and hair cycling [143].

## 10. The Therapeutic Role of Exosomes in Cutaneous Diseases

**Figure 4:**
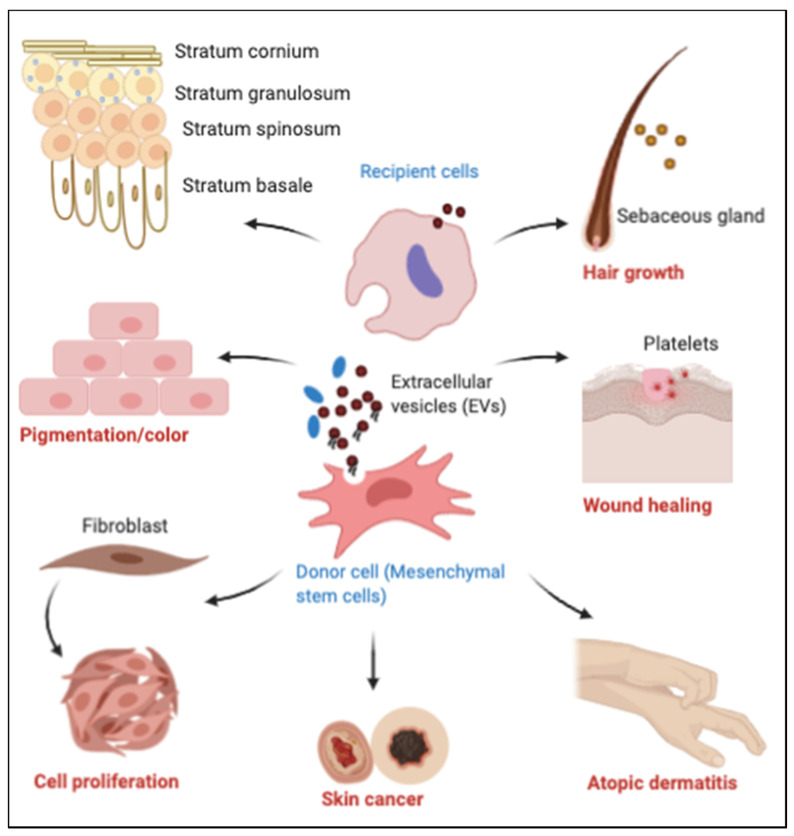
Therapeutic role of EVs (exosomes) in cutaneous diseases. Exosomes with specific cargo can attenuate deregulated changes associated with pathogenesis of different skin diseases such as wound healing, hair problems, cell proliferation, pigmentation and cancer development.

Exosomes have been given special attention as therapeutic and diagnostic targets due to their capacity as carriers of almost all extracellular biological modulators of inflammation, autoimmunity and cancer. Due to their structure, content and ability to fuse with the target cells, exosomes-controlling agents may be promising for drug development in cutaneous diseases (Figure 4), including skin cancers [74,150,151,152,153,154,155,156,157]. Along that line, recent findings suggest a potential prognostic and therapeutic importance of exosomes in various cancer types including prostate cancer [158], lung cancer [159], head and neck cancer [160], breast cancer [161] and colorectal cancer [162]. Exosomes are also implicated in metabolic and cardiovascular diseases [163] and stroke [164] morbidities which are also associated with an increased risk in psoriasis patients. Consequently, one may anticipate that targeting exosomes in psoriasis may also have a beneficial effect for the treatment of its comorbidities, and probably vice versa.

In recent years, exosomes, due to their unique features and presence in various types of body fluids, have gained enormous scientific interest as potential ‘biomarkers’ for various diseases including skin [151,165,166,167]. A number of clinical trials on exosomes further support their therapeutic importance [168]. Managing skin wounds due to various factors including burns, trauma or ulcers is a major health and socio economic concern and thorough investigation on underlying mechanisms is required [169,170]. Considering the complications associated with available therapeutic majors of skin wounds, role of exosomes has gained enormous attention at different steps of drug research and development. Interestingly, exosomes derived from MSCs have been shown to possess better therapeutic potential for wound management as these cells are vital in various steps of cutaneous regeneration at the wound site via interacting with a number cell types, signaling pathways and growth factors etc., related to tissue regrowth and development [169,170,171]. The stem cells-derived EVs help coordinate wound repair in a paracrine manner by facilitating transfer of transcription factors, growth factors as well as anti-inflammatory factors [172]. Recently, Li et al. demonstrated that exosomes derived from adipose-derived stem cells play critical role in augmenting wound repair and healing mechanisms due to diabetic foot ulcers (DFU). Further, stem cells-derived exosomes, with upregulated nuclear factor erythroid 2-related factor 2 (NRF2), exert therapeutic action via alleviating inflammation and oxidative stress, with increased granulation tissue formation, angiogenesis and levels of growth factor expression, and hence these can be suitable for the treatment of DFUs [173]. Moreover, it has also been shown that exosomes derived from amniotic fluids potentiate wound healing and inhibit scar formation via promoting migration and proliferation of fibroblasts [174]. Studies have been performed to elucidate the underlying signaling mechanisms of exosomes-mediated wound repair which is critical for the development of therapeutics. For instance, roe of AKT/PI3K, WNT etc., among others have been reported in exosome mediated wound healing [175,176]. Moreover, Fang et al., show that the MSCs derived exosomes enriched with microRNAs play key role in preventing scars formation during wound healing by suppressing myofibroblast accumulation via inhibiting the transforming growth factor-β2/SMAD2 pathway [177]. Another report showed that exosomes derived from fetal dermal mesenchymal stem cells (FDMSCs) play major role in wound healing via stimulating enhanced proliferation, migration, and secretion of fibroblasts by targeting notch signaling [178]. Overall, based on the available findings it can be suggested that exosomes are vital not only in pathogenesis but also in therapy of cutaneous wounds and have great potential of a promising biomarker.

Exosomes, due to their myriad features in different biological mechanisms including in the antigen presentation and immune regulations, seem to play significant role in the development of autoimmune diseases. Therefore, they are of great therapeutic importance both at diagnostic and therapy level for various human diseases, including autoimmune connective tissue diseases (ACTD) [179]. Targeting exosomes can also be a strategy to manage skin autoimmune diseases, including psoriasis and sclerosis. For example, Nakamura et al. demonstrated that low level of exosomes in systemic sclerosis (SSc) patients suppress prolonged wound healing via down-regulation of collagen, thus leading to increased risk of developing pitting scars and/or ulcers. Moreover, they further reported that enhanced exosomal level stimulated wound healing [180]. In this context, a number of reports revealed the critical role of exosomes in the pathogenesis of systemic sclerosis as they are critically related with immunity, vascular damage, and fibrosis [181]. Exosomes from myeloid-derived suppressor cells (MDSC) have exhibited significant therapeutic potential for alopecia areata, an autoimmune disorder associated with hair loss [182]. There is also evidence about critical role of exosomes in the pathogenesis of psoriasis, another cutaneous autoimmune disease, and hence exosomes may provide a novel and effective therapeutic tool [128,129,183]. Bullous pemphigoid is an autoimmune inflammatory disorder associated with dermal- blister formation and recently Fang et al. observed that exosomes from blister fluids of patients with bullous pemphigoid, play critical role in its pathogenesis by inducing inflammatory response due to over production of cytokines and chemokines via modulating ERK1/2 and STAT3 signaling [184].

Adipose derived stem cells exosomes (AD-exos) with upregulated miR-21 expression attenuate wound healing in rodents and improve the migration and proliferation of HaCaT cells by modulating MMP-2 and TIMP-1 expression through PI3K/AKT signaling [185]. Differentially expressed proteins of serum exosomes from burn patients modulate expression of proteins regulating enzyme inhibitor activities, heparin-binding, coagulation and lipid transport. Further, upregulated ITGA2B and ITGB3 expression in serum of burn patients play major role in PI3K/AKT mediated injury detection and repair [186]. Therapeutic potential and the underlying mechanism of human umbilical cord MSC-derived exosome (hucMSC-Ex) mediated cutaneous wound healing has been evaluated in rat burn model with results suggesting upregulated Wnt/β-catenin-mediated wound re-epithelialization and cell proliferation [187]. Moreover, there is some clinical data on the therapeutic role of bone marrow-derived mesenchymal stem cells (BM-MSCs) in the management of recessive dystrophic epidermolysis bullosa (RDEB), a severe blistering disease due to lack of type VII collagen production inside human body. McBride et al. demonstrated that BM-MSC-derived EVs play major role in the upregulation of type VII collagen level via directly donating BM-MSC type VII collagen protein and also by inducing translation of VII collagen protein in the recipient cells [188].

In summary, exosomes, due to virtue of their complex contents, play integral role in wound management such as healing and repair via modulating various signaling mechanisms and hence could be of great therapeutic importance (Table 1).

## 11. Conclusions

Through the detailed discussion presented above, we have highlighted the clinical importance of exosomes in cutaneous diseases. Exosomes serve as cargo for proteins, nucleic acids, lipids etc. They are critical for cellular interaction/communication and thus associated with transfer of vital information or moieties critical in biological homeostasis and also in disease pathogenesis. Hence, exosomes are agents of clinical relevance, relevant for diagnosis as well as targeted therapy.

There has been progress in our understanding of how the exosomes are critical in cutaneous disease and therapy, thorough collaborative investigations focusing on precise isolation techniques and characterization of exosomes and their contents. However, detailed knowledge of the underlying mechanisms and mode of transport and cell interaction is imperative in order to develop novel and effective approach for therapy, diagnosis and disease prognosis. Moreover, exploring the exact mechanism(s) by which exosomal contents (e.g., proteins, lipids, nucleic acid etc.) modulate genes related to skin inflammation and immunity (e.g., cytokines, chemokines, NF-κB etc.) would further strengthen the clinical significance of EVs, particularly in cutaneous diseases. Although recent developments suggest vital role of stem cells derived Evs in cutaneous disease pathogenesis and therapy including drug resistance, the mechanisms generally remain poorly understood. Finally, considering the important novel therapeutic features of Evs, focus should be on clinical trials, within the scope of individual cutaneous diseases, to take the knowledge from benchside to bedside.

## Figures and Tables

**Table 1:** Role of EVs in cutaneous diseases.

Cutaneous Disease Type	Exosome or Extracellular Vesicle	Underlying Mechanisms	Biomarkers/Associated Proteins/RNAs	References
Psoriasis	Psoriatic arthritis (PsA) derived exosomes, apoptotic bodies	Enhancement of osteoclastogenesis	let-7b-5p, miR-30e-5p	[189,190]
	IL-17A producing exosomes	T-cell activator mediated innate immunity and contributing to pathogenesis of inflammation	CD1a autoreactive T cells	[126,127]
	Plasma derived exosomes	Development of immune diseases and bone metallic dysfunction	has-miR-151a-3p, has-miR-199a-5p, has-mimR-370-3p	[133]
Squamous cell carcinoma (SCC)	Oral SCC derived EVs	Potential biomarkers	miR-512-3p, miR-412-3p	[191]
	CAF derived exosomes	Predictor of cisplatin resistance	miR-196a	[192]
	Serum derived exosomes	Valuable biomarker	HOTAIR, miR-21	[98,193]
Alopecia areata	Myeloid derived suppressor cells (MDSC) exosomes	Partial hair regrowth and progression prevention	FoxP3, arginase 1	[182]
Bullous pemphigoid	Keratinocyte EVs	Inflammatory and immune responses	CD63, CD81, CD9	[184]
	Blister fluid derived exosomes	Inflammatory and immune responses	TNF-a, CXCL8 and IL-6	[12,184]
Melanoma	NK-92 cells derived exosomes	Cytotoxic effects against melanoma	FasL, ALIX, CD63	[12]
	T-cells derived EVs	Prolonged progression free survival and overall improved survival	PD-1 and CD28	[12,96]
	Plasma of metastasis melanoma EVs	Useful biomarkers for disease progression	S100B, miR-17, -19a, -21, -126 and -149.	[16]
	EVs	Gadd45b, WNT	miR-300, miR-149,miR-211, let7a etc.	[95,96]
	Melanoma derived exosomes	Biomarker for Increase in cell invasion and migration	CD-81	[194]
Systematic sclerosis (SS)	SS fibroblast derived exosomes	Acceleration of skin healing	CD63, CD9, CD81	[180,181]
	Exosomes isolates from SS serum	Apoptosis and collagen expression regulation	let-7g, miR-23b, miR-17, miR-29a	[181,195]
Atopic dermatitis (AD)	Adipose tissue (AT)—derived exosomes	Cell-free therapy, relieve from AD symptoms	CD86+ and CD206+ cells	[144]
	Fungi-derived exosome like vesicles	Skin pH regulation	Inducing TNFa and IL-4 responses	[12,196]
Wound healing	MSCs derived EVs	Skin repair, cell migration, restoring skin integrity	miR-205	[197]
	Adipose mesenchymal stem cells (ASCs) exosomes	Tissue regeneration, cell migration, collagen synthesis, proliferation	cyclin-1, N-cadherin, collagen I, III, PCNA	[198]
	LPS-pretreated hUC-dMSCs EVs	Anti-inflammatory properties	miRNA let-7b	[169]
